# Psychometric Properties of the Greek Version of the Inpatient Dignity Scale

**DOI:** 10.3390/healthcare14070855

**Published:** 2026-03-27

**Authors:** Maria Gkarliaridou, Vasiliki Matziou, Sofia Zyga, Evangelos Fradelos, Maria Polikandrioti, Victoria Alikari

**Affiliations:** 1 Post Graduate Program “Applied Nursing Science-Nursing of Renal Diseases”, Department of Nursing, National and Kapodistrian University of Athens, 10561 Athens, Greece; gkarliaridou@gmail.com (M.G.); vmatziou@nurs.uoa.gr (V.M.); 2 Department of Nursing, University of Peloponnese, 22100 Tripolis, Greece; zygas@uop.gr; 3 Department of Nursing, University of Thessaly, 41500 Larisa, Greece; efradelos@uth.gr; 4 Department of Nursing, University of West Attica, 12243 Athens, Greece; mpolyk@uniwa.gr

**Keywords:** dignity, patients, reliability, validity, scale

## Abstract

**Background/Objectives:** Patient dignity is one of the central values in nursing, equivalent to justice, freedom, and individuality. The purpose of this study was to investigate the psychometric properties of the Greek version of the Inpatient Dignity Scale (IPDS). **Methods:** In this descriptive, cross-sectional study, 280 patients from three Hemodialysis Units (HD) completed the IPDS, a self-completed questionnaire assessing patients’ expectations regarding dignity and patients’ satisfaction with dignity. Items are categorized into four dimensions, both for expectations and satisfaction: Respect as a Human Being, Respect for Personal Feeling and Time, Respect for Privacy, and Respect for Autonomy. For the translation into Greek, a double forward-backward translation process was followed, and subsequently, cultural adaptation was carried out. Construct validity was tested using the Confirmatory Factor Analysis (CFA) conducted in AMOS 26.0. Convergent validity was assessed through correlations with the Caring Behaviors Inventory-16 (CBI-16) and correlations between the dimensions of the IPDS. Repeatability was assessed using the Intraclass Correlation Coefficient (ICC), and internal consistency using Cronbach’s alpha. The SPSS 26.0 statistical program was used for the descriptive and correlational analyses (*p* < 0.05). **Results:** The mean age of participants was 64.8 years old. CFA revealed an acceptable fit for the questionnaire (CFI 0.92–0.93, TLI 0.91–0.94, and RMSEA < 0.08 for both expectations and satisfaction). The IPDS was significantly and positively correlated with the CΒΙ-16, indicating good convergent validity. Cronbach’s alpha was >0.70 in all dimensions of the IPDS, indicating good internal consistency. **Conclusions:** The Greek version of IPDS is a valid and reliable tool to measure patients’ perceived dignity.

## 1. Introduction

The concept of human dignity has been explored since the beginning of the 21st century in a universal and multidimensional manner [1]. Through the contemporary literature, characteristics of patient dignity are recognized, including respect for their autonomy and personal life, the confidentiality of sensitive data (both personal and medical), therapeutic interactions with nursing staff, and acceptance of patients’ ethnicity, age, gender, religious beliefs, and cultural background [2].

The World Health Organization (WHO) recognizes human dignity as one of its core principles, equal to justice, equality, and independence [3]. In the field of nursing science, the concept of dignity was introduced by Shotton and Seedhouse (1998) [4]. The International Council of Nursing (ICN) recognizes human dignity as a fundamental principle of nursing [5]. Since the era of Florence Nightingale, nurses have cared for patients with dignity, respecting both the patients themselves and their personal privacy [6]. In bioethics, humanity has been confronted with new concepts such as death with dignity, disconnection from the life-support machine of comatose patients, organ transplantation, euthanasia, cloning, and, therefore, dilemmas about whether dignity functions in an evolutionary or restrictive manner [7].

Research on dignity in the provision of care in hospitals and nursing homes initially focused on palliative care at the end of life and has now expanded to patients suffering from chronic diseases [8]. Patients undergoing hemodialysis (HD) represent an exceptionally sensitive population due to the chronicity of the disease and the nature of the treatment. Frequent dependence on healthcare professionals, the hemodialysis machine, technology, and medications can affect the sense of autonomy. In addition, repeated invasive procedures (such as catheterizations and central venous catheter placement) and uncertainty about the future may affect perceived self-image [9]. In this regard, patients undergoing HD constitute a population in which issues of dignity are particularly salient. Patients undergoing HD suffer from a variety of comorbidities, such as heart failure and diabetes mellitus, among others. Possible complications related to vascular access, pain, discomfort, vulnerability, and dependence on the dialysis machine further diminish patients’ health-related quality of life and, consequently, their self-perceived dignity [9,10]. This confronts health professionals with additional issues and challenges, such as the conservative management of chronic kidney disease and the patients’ right to withdraw from HD. Palliative care for patients undergoing HD is integrated into renal nursing care when life expectancy is limited, and HD is unlikely to improve patients’ overall health in the long term. Such a decision preserves patients’ inherent dignity by allowing them to exercise autonomy and self-determination in managing their care [11].

Due to the close relationship of patients undergoing hemodialysis with healthcare professionals, perceived dignity is often threatened as patients undergoing HD are treated as part of the routine in daily clinical practice rather than as persons with their individualized needs. A typical example is the use of the term “dialysis patient” instead of addressing them by their names. As a result, patients experience stigmatization and the undermining of human dignity. This sequence of reactions negatively affects their psychosocial well-being and their trust in the health care system [12,13].

Measuring perceived dignity requires the use of reliable instruments, especially among vulnerable populations at increased risk of dignity impairment, such as patients undergoing hemodialysis. The majority of these scales have been used in elderly populations, individuals in long-term care facilities, and individuals receiving home palliative care [14]. The Patient Dignity Inventory explores key dimensions related to dignity distress, dependence, and social support [15]. The Attributed Dignity Scale, designed for older adults, examines self-esteem, self- respect, and respect toward others. The Measurement Instrument for Dignity AMsterdam-for Long-Term Care Facilities assesses determinants influencing individuals’ dignity in long-term care facilities [14]. The Palliative Patient Dignity Scale is intended for patients receiving home palliative care at the end of life and their caregivers [15]. Finally, the Hospitalized Older Adults’ Dignity Scale is administered to older adults during their hospitalization. It assesses the patient’s participation in clinical decision-making, interactions with healthcare professionals, and the respect received [16]. Nevertheless, the extent to which dignity is thoroughly assessed in patients undergoing HD remains unclear.

Previous studies on the reliability and validity of dignity scales have demonstrated good psychometric properties and largely consistent factor structures across populations. However, most studies have been conducted among general patient populations, and there is limited data on patients undergoing HD. In the present study, the Inpatient Dignity Scale (IPDS) was used in Greek patients undergoing HD to fill this research gap. This specific scale was chosen because it assesses dignity from two perspectives: patients’ expectations and satisfaction. The aim of the study was to translate, culturally adapt and study the construct validity and internal consistency of the IPDS among patients undergoing HD.

## 2. Materials and Methods

This study employed a descriptive, cross-sectional, and correlational design and included the assessment of psychometric properties of the scale, particularly its internal consistency and validity. The study was conducted in two phases: (1) the first phase included the translation and cultural adaptation of the scale; (2) the second phase included the assessment of the validity, reliability, and internal consistency of the scale.

### 2.1. The Sample and Settings

The research sample consisted of patients undergoing HD at three HD of public general hospitals (“Naval” Hospital of Athens, General Hospital of Athens “Laiko”, and General Hospital of Athens “Ippokrateio”), in Athens (the capital and the most densely populated city in Greece). The selection of these HD was based on the researchers’ ease of access, as they have a professional position (convenience sample). These three hospitals were chosen for practical reasons. Specifically, they are two different public care settings (two public general hospitals and one military hospital), providing a diverse sample of patients undergoing dialysis. The inclusion criteria were: patients undergoing HD three times a week, able to read, write, and understand the Greek language, being capable of reading and signing the informed consent form, and being oriented to time and place. Patients with cognitive or psychological disorders, visual or hearing impairments, or limited self-care capabilities were excluded from the study. Data regarding the cognitive and psychological status, as well as eye and ear health, of patients were obtained from the patients’ health files. Of the total 329 patients undergoing HD (during the research period), 311 were eligible, and 280 provided written consent. The response rate was 90.0% among the eligible patients. The size of the sample (N = 280), according to the recommendation of at least 10 participants per item [17], indicates its adequacy for psychometric analysis. Questionnaires were distributed by the researchers and were completed between the 2nd and 3rd hour of the HD session. The study was carried out between March and November 2023.

### 2.2. Instruments

Data were collected using the Inpatient Dignity Scale (IPDS), a self-administered questionnaire developed in 2019 for hospitalized inpatients in Japan and the United Kingdom [18]. The scale assesses patients’ perceived dignity in two sections: expectations (16 items) and satisfaction (18 items). For the expectations section, the items are categorized into four dimensions of dignity: (a) Respect as a Human Being (items 1–6); (b) Respect for Personal Feeling and Time (items 7–9, 13, 14); (c) Respect for Privacy (items 19–21); and (d) Respect for Autonomy (items 11, 12). For the satisfaction section, the items are also grouped in four dimensions: (a) Respect as a Human Being (items 1–6); (b) Respect for Personal Feeling and Time (items 8–10, 14–18); (c) Respect for Privacy (items 19, 21); and (d) Respect for Autonomy (items 11, 12). Patients respond to a 5-point Likert scale both for expectations (“How strong are your expectations?”, 1 = “not at all” to 5 = “very strong expectations”) and for satisfaction (“How satisfied are you?”, 1 = “very dissatisfied” to 5 = “very satisfied”). The score is calculated by summing the corresponding items and dividing by their total number. The higher the score, the higher the level of perceived dignity. There is no cut-off score. Psychometric properties (validity, reliability, and internal consistency) of the original version are good, with Cronbach’s alpha for the expectations ranging from 0.72 to 0.88 and for the satisfaction ranging from 0.72 to 0.90 [18]. The IPDS has been translated into Mandarin Chinese [19] and into Spanish [20] with excellent internal consistency (Cronbach’s alpha 0.820 for the expectations and 0.995 for the satisfaction section). The scale can be applied to community patients, hospitalized patients receiving daily care, and palliative care patients [14].

The Caring Behaviors Inventory-16 (CBI-16) [20] assesses the caring behaviors as perceived by patients and nurses. For this study, the version of CBI-16 was administered to patients. It consists of 16 items rated on a 6-point Likert scale, with scores ranging from 1 (never) to 6 (always); lower scores indicate less desirable behavior and vice versa. The CBI-16 is a brief, easy-to-administer instrument with excellent internal consistency (Cronbach’s alpha = 0.967) [20]. The Greek version of the CBI-16 was used to study the convergent validity of the IPDS [21].

Sociodemographic and clinical data were also recorded: age, gender, marital status, educational level, occupational status, place of residence, dialysis access, and years on HD.

### 2.3. Translation of the IPDS

The ΙPDS scale was translated from English into Greek, following the guidelines of the International Society for Pharmacoeconomics and Outcomes Research (ISPOR) Task Force for Translation and Cultural Adaptation [22]. Specifically, the double forward-backward translation procedure was employed. Initially, two independent bilingual health professionals translated the English version (source language) of the scale into Greek (target language) (forward translation). The principal investigator of the study reviewed the two Greek versions and produced a third Greek version. This process resulted in the final Greek version of the scale (first reconciliation version). Subsequently, the Greek version was back-translated into English by two different translators (a bilingual health professional and an English-language professor) who had not read the original English version (backward translation). Following the comparison of the two English versions of the questionnaire, semantic equivalence between them was verified (second reconciliation version). The final Greek version of the scale was also reviewed by a panel of bilingual experts in nursing.

### 2.4. Cultural Adaptation of the IPDS

For the cultural adaptation of the IPDS, the cognitive interview process was applied. According to the literature [22], a sample of 15 participants is sufficient for the process. The participants then completed the General Impression Instrument, through which they expressed their overall opinions about the scale. Participants were asked to indicate whether any items were unclear and difficult to understand. In cases where ambiguities were identified, they suggested alternative wording without changing the meaning of each question. The researchers incorporated the participants’ suggestions into the second reconciliation version, from which the final Greek version of the IPDS emerged. In total, 13 out of 15 (86.6%) participants reported no difficulty in understanding the scale items; one participant stated that the items were difficult to understand, and one participant stated that the items were moderately understandable.

### 2.5. Reliability of the IPDS

For the reliability analysis, the test–retest method was applied. Specifically, 40 participants completed the scale twice with an interval of two weeks. The time interval between the two administrations was used to minimize the possibility that participants would recall their answers from the first administration [23].

### 2.6. Statistical Analysis

The distributions of the quantitative variables were tested for normality using the Kolmogorov–Smirnov test. For those that were normally distributed, the means and standard deviations were used to describe them, while for those that were not normally distributed, medians and interquartile ranges were used. Both parametric and non-parametric descriptive statistics were included to offer a complete summary of the data distribution. Absolute (N) and relative (%) frequencies were used to describe the qualitative variables. The Spearman correlation coefficient (rho) was used to test the relationship between two quantitative variables. Confirmatory factor analysis (CFA) with maximum likelihood estimation was used to test the construct validity and confirm the factor structure of the IPDS. Several approaches were used to assess the fit of the confirmatory factor analysis model, including the CFI (Comparative Fit Index), TLI (Tucker–Lewis Index), RMSEA (Root Mean Square Error of Approximation), and SRMR (Standardized Root Mean Squared Residual). According to established guidelines, the CFI and TLI indices can take values from 0 to 1, and values close to or above 0.90 indicate good model fit, while values close to or above 0.95 suggest excellent fit. The CFI index is considered more suitable for model estimation as it considers the sample size. RMSEA values below 0.05 indicate good fit, and values up to 0.08 indicate acceptable fit. The SRMR index value < 0.08 is generally considered a good fit.

The internal consistency of the questionnaire was tested using Cronbach’s alpha. Intra-class Correlation Coefficients (ICCs) were used to investigate the agreement of the responses in the test–retest process. Agreement was considered low for ICC ≤ 0.40, moderate for ICC 0.41–0.60, high for ICC from 0.61 to 0.80, and very high for ICC > 0.80. The significance levels were two-sided, and statistical significance was set at 0.05. SPSS version 26.0 (IBM Corp., Armonk, NY, USA) was used for descriptive and correlational analyses, and AMOS 26.0 (IBM Corp., Armonk, NY, USA) for confirmatory factor analysis.

### 2.7. Ethics

The study protocol was approved by the Ethics Committee of the Department of Nursing of the National and Kapodistrian University of Athens (apr. number 419/10.10.2022), as well as by the scientific councils of the hospitals where the study was conducted: The Naval Hospital of Athens (apr. number 7/22/26.10.2022), the General Hospital of Athens “Laiko” (27.10.2022) and the General Hospital of Athens “Ippokrateio” (apr. number 26/13.12.2022).

This research met the fundamental ethical principles governing research. Confidentiality was strictly maintained regarding patients’ information, and data were stored securely. The anonymity was guaranteed as questionnaires were submitted to the researchers in closed envelopes. The results obtained were used solely for this study by the research team.

Permission to use the IPDS was not required, as stated by the scale’s developers in the relevant article on reliability and validity [18].

## 3. Results

### 3.1. Demographic Characteristics of the Sample

The sample consisted of 280 patients undergoing HD with a mean age of 64.8 years (SD ± 13.3 years). The demographic and clinical data of the participants are given below (Table 1).

### 3.2. Descriptive Statistics of the Expectations and Satisfaction Sections of the IPDS

Table 2 presents the descriptive statistics of the expectations and satisfaction sections of the IPDS. The possible range of scores was from 1 to 5, with higher values indicating higher respect for the respective domain. The highest mean score of expectations was observed in the dimension “Respect as a Human Being” (4.7 ± 0.4) and the lowest in the dimension “Respect for privacy” (4.3 ± 0.9). The highest mean score of satisfaction was observed in the dimension “Respect as a Human Being” (4.5 ± 0.5) and the lowest in the dimension “Respect for autonomy” (4 ± 0.8).

### 3.3. Construct Validity of the IPDS

To test structural validity, Confirmatory Factor Analysis (CFA) was conducted for both expectations and satisfaction of the IPDS. CFA revealed an acceptable fit for the questionnaire, with CFI 0.92 for expectations and 0.93 for satisfaction, TLI 0.91 for expectations and 0.94 for satisfaction, RMSEA index below 0.08 for both expectations and satisfaction, and SRMR < 0.08 for both expectations and satisfaction. The standardized factor loadings were satisfactory for all items, ranging from 0.50 to 0.62, supporting the four-factor model. Τhe Composite Reliability (CR) values for the four factors ranged from 0.60 to 0.70 for both expectations and satisfaction, demonstrating moderate reliability, which is considered acceptable for psychometric evaluation of scales. Furthermore, the χ^2^/df ratio was 4, indicating an acceptable to good model fit. The factorial structure that emerged in the present study aligns with the original theoretical structure of the IPDS questionnaire, as defined by the instrument’s developers. The following table (Table 3) gives the results of the CFA.

### 3.4. Convergent Validity

For the study of convergent validity, a comparison was made between the IPDS and the CBI-16. The sections of the IPDS, regarding participants’ expectations and satisfaction with dignity, were significantly and positively correlated with the CΒΙ-16. Specifically, the r values for expectations ranged from 0.18 (*p* ≤ 0.003) to 0.40 (*p* ≤ 0.001), while for satisfaction from 0.30 (*p* ≤ 0.001) to 0.58 (*p* < 0.001). The strongest correlations were observed in the dimensions “Respect for Personal Feeling and Time” and “Respect as a Human Being”.

In addition, the correlations between the dimensions of the IPDS, regarding participants’ expectations and satisfaction, were all positive and statistically significant (*p* < 0.001). The highest r-value was 0.72, and the lowest was 0.55 for both expectations and satisfaction, indicating that the four factors are parts of the same structure (Table 4).

### 3.5. Reliability and Internal Consistency of IPDS

To assess repeatability, the test–retest procedure was used, and the Intraclass Correlation Coefficient (ICC) was calculated in a sample of 40 participants. Results are presented in Table 5. Substantial agreement was observed across all items of the IPDS, encompassing both the expectation and satisfaction sections, indicating that the instrument’s measurement reliability is acceptable.

The table below (Table 6) presents the Cronbach’s alpha coefficient for each dimension when a single item is deleted, along with the item-total correlation for each question. Notably, the item-total correlation for Item 13 was negative, and the Cronbach’s alpha of the scale improves if this item is removed (Item 13 Corrected Item-Total Correlation: −0.050; Cronbach’s Alpha including item 13: 0.45, Cronbach’s Alpha if Item 13 deleted: 0.71). Thus, with the removal of item 13, the Expectations section comprises 15 items. The remaining correlation coefficients between each item and the scale dimensions were considered satisfactory. Cronbach’s alpha index was >0.70 in all dimensions of the IPDS, indicating that the IPDS has good internal consistency. Cronbach’s alpha for the Expectations section was 0.82, and for the Satisfaction section was 0.90.

## 4. Discussion

This study aimed to investigate the psychometric properties of the IPDS among patients from three general hospitals in Athens. It is the first study to use the IPDS in Greece, and particularly in patients undergoing HD. The questionnaire was translated and culturally adapted for Greek patients undergoing HD, and subsequently, its validity and reliability were evaluated. According to the Questionnaire Comprehension and Evaluation Tool, the instrument was easy for patients to understand and complete.

Japanese researchers [18] developed the IPDS to measure hospitalized patients’ expectations and satisfaction with perceived dignity in daily care among Japanese and British hospitalized patients. Then, Huang et al. [19] translated and culturally adapted the IPDS into Mandarin Chinese for hospitalized patients in tertiary hospitals in China, and Merino et al. [20] evaluated the psychometric properties of the scale among Spanish hospitalized patients. Given that the psychometric properties of the scale have been validated solely within the aforementioned cultures, comparisons of the findings of the present study are therefore limited to those studies.

Regarding the structural validity of the scale, this study demonstrated the maintenance of the original structure of the IPDS in the Greek population of patients undergoing hemodialysis. Specifically, the factor analysis led to the extraction of 4 factors: Respect as a Human Being, Respect for Personal Feeling and Time, Respect for Privacy, and Respect for Autonomy. These four factors that emerged in the Greek version of the IPDS are composed of the same groups of questions that made up the original Japanese version of the tool [18], although the scale was developed in a Japanese cultural environment, with different socio-cultural characteristics. Also, the same structural model was found in the study of the factor analysis of the Spanish version of the IPDS [20]. This finding is particularly interesting, given that the concept of dignity is considered to be partly culturally dependent and may be influenced by social values, perceptions of health care, and different communication patterns. A possible interpretation of the similarity is that dignity may have a common, cross-cultural core meaning and is a fundamental and universal element of the human experience of health care [24]. Regardless of the illness, hospitalized patients experience loss of control, dependence on staff and the health care system, as well as changes in their social identity and autonomy. These shared experiences may lead to the emergence of similar psychosocial needs and priorities in relation to maintaining dignity [25]. Furthermore, the careful translation and cultural adaptation process applied in this work likely contributed to the preservation of the conceptual features of the original version. This consistency demonstrates the stability of the scale’s conceptual structure and reinforces its cross-cultural validity. This fact suggests that the clinical dimensions of dignity are recognized in the same way by patients.

In contrast, the Mandarin version of the IPDS [19] scale presented a five-factor structure, which may be related to cultural specificities. Although dignity may be viewed as a global concept, in Chinese culture, values of respect and an emphasis on social roles tend to provide greater differentiation to individual dimensions of dignity [26].

To study the convergent validity, correlations were tested between the dignity scale and the CBI-16 scale. Significant positive correlations were observed, as high levels of respect for the person, respect for personal time, privacy, and autonomy were associated with higher levels of caring behaviors. This finding is consistent with the original study by Ota et al. [18], which reported positive correlations between the IPDS and self-esteem, as measured by the Rosenberg Self-Esteem Scale. In contrast, significant but negative correlations between the IPDS and the PDI scale were found in the study of the Mandarin version of the IPDS [19]. Although the correlations with the CBI-16 were statistically significant, their relatively low strength for some dimensions suggests that the dimensions interpreted by the two instruments are related but not equivalent. This finding may indicate conceptual variances between perceived dignity and perceptions of patients’ caring behaviors, particularly in patients undergoing HD. Thus, dignity represents a wider concept that is not completely reflected by measures of caring behaviors alone [26].

The correlations between the dimensions of the IPDS, regarding participants’ expectations and satisfaction, were all positive and statistically significant. As far as expectations are concerned, higher respect for personal emotion and time, privacy, and autonomy were related to higher respect for human beings, while respect for privacy and autonomy was also positively related to respect for personal emotion and time. These strong positive correlations support the assumption that the four dimensions of the IPDS are parts of the same structure.

Regarding reliability, the test–retest procedure demonstrated good agreement between the two measurements for almost all items of the IPDS in both the sections of expectations and satisfaction with dignity, indicating acceptable measurement reliability.

The internal consistency of the IPDS in this study was considered satisfactory. Specifically, the total Cronbach’s alpha was 0.82 and 0.90 for expectations and satisfaction of the IPDS, respectively. As far as the particular dimensions of “Respect as a Human Being,” “Respect for Personal Feelings and Time,” “Respect for Privacy,” and “Respect for Autonomy,” Cronbach’s alpha ranged from 0.70 to 0.79 for both expectations and satisfaction domains. This finding aligns with the study of Mandarin [19] and Spanish [20] validation of the scale among hospitalized patients, where internal consistency was good [19].

An important finding of the present study concerns Item 13 (“Nurse/physicians of my gender give me care”) of the IPDS. Item 13 was included within the Expectations domain in both the Japanese [18] and Spanish [20] versions of the IPDS but not in the satisfaction section. However, in this study, this item demonstrated a negative correlation with the remaining items and did not contribute to the internal consistency of either the Expectations or Satisfaction sections, although the four-factor structure of the scale was preserved. Consequently, Item 13 was removed, resulting in a significant improvement in the internal consistency of the scale. This finding is likely due to the specific cultural characteristics of the Greek population, bringing to the surface critical issues in the cross-cultural adaptation of psychometric tools. Item 13 is related to the patient’s preferences for care provided by health professionals of the same or different gender. Such preferences may be interpreted differently across cultures and, especially, within a population of patients undergoing HD, who often develop long-term and close relationships with healthcare professionals [27]. Furthermore, the removal of Item 13 may affect comparison with other validated versions of the IPDS, in which this item was retained. This implies that some items may play a different role in diverse social settings and highlights the necessity for careful re-evaluation when culturally adapting psychometric tools and comparing factor structures across populations.

In the present study, patients undergoing HD maintained relatively high levels of satisfaction with dignity across all dignity factors of the IPDS. Specifically, higher mean scores were observed in the dimension of “Respect as a Human Being” (mean 4.7) and lower in the dimension “Respect for Privacy” with a mean of 4.3 points. These findings are consistent with the results on expected dignity levels reported in the multicenter study conducted in hospitals in Japan, Singapore, and the United Kingdom [18] during the development of the original IPDS.

Patients’ levels of satisfaction with perceived dignity remained relatively high across all four IPDS factors, with higher mean scores on Respect as a Human Being and lower mean scores on Respect for Autonomy. This finding is consistent with previous studies in hospitalized patients [18,20,28]. This variation may suggest that basic needs for respect for human beings are more satisfied than needs for autonomy, which may guide nursing interventions to enhance perceptions of dignity across all dimensions [29].

The survey has some limitations that should be acknowledged. Data were collected during HD sessions. Therefore, the presence of other patients and healthcare professionals may have affected the objectivity of the responses. The use of a convenience sample, as the study was conducted in the HD of three hospitals in Athens, introduces potential selection bias and limits the generalizability of the results. Therefore, the findings may not be fully representative of the broader Greek healthcare settings. Due to the cross-sectional nature of the study, a causal relationship between the variables cannot be established. Also, possible response bias cannot be ruled out, as patients with more positive experiences may have participated more willingly. The use of self-reported questionnaires may have been affected by social desirability bias, as patients may have avoided negative evaluations of staff.

A strength of the present study is that, to our knowledge, it is the first time that the psychometric properties of a dignity scale in patients under HD have been studied in Greece.

## 5. Conclusions

The Greek version of the IPDS demonstrates good structural and convergent validity, reliability, and internal consistency. It retains a stable four-factor structure and is a useful tool for exploring patients’ levels of expectations and satisfaction regarding dignity among Greek patients undergoing HD. This study highlights the importance of reliable assessment of patients’ perceived dignity not only at the clinical but also at the health policy level. The Greek version of the IPDS may support the assessment of care quality and ethical aspects in the context of patient-centered care, as well as the design and improvement of health services.

Future research could investigate the scale’s applicability across diverse patient populations, including hospitalized and outpatient settings, and across both chronic and acute illnesses. This would allow for comparisons across populations and more robust conclusions to be drawn.

## Figures and Tables

**Table 1:** Demographic and clinical data of participants (N = 280).

		Ν	% ^3^
Gender	Male	180	64.2
	Female	100	35.7
Place of residence	Rural	25	8.92
	Semi-urban	30	10.7
	Urban	225	80.3
Marital status	Unmarried	48	17.1
	Married	141	50.3
	Divorced	26	9.2
	Widower/widow	65	23.2
Living alone	No	211	75.3
	Yes	69	24.6
Educational level	Illiterate	1	0.35
	Elementary School	64	22.8
	Graduate Middle School	41	14.6
	High School Graduate	95	33.9
	University Student	13	4.64
	University Graduate	66	23.5
Occupational status	Unemployed	13	4.64
	Household	44	15.7
	Self-employed	10	3.57
	Private employee	14	5.0
	Public employee	15	5.35
	Retired	175	62.5
	Other	9	3.2
Dialysis access	Fistula	121	43.2
	Central venous catheter	41	14.6
	Graft	118	42.1
Age	Mean (SD) ^1^	Median (IQR) ^2^	
	64.8 (13.3)	68.0 (56.0–76.0)	
Years on HD	6.3 (5.2)	6.0 (2.0–8.0)	

^1^ Standard Deviation, ^2^ Interquartile Range, ^3^ Percentages may not sum to 100% due to missing data/rounding.

**Table 2:** Descriptive characteristics of the Inpatient Dignity Scale (IPDS).

	Min	Max	Mean (SD) ^1^	Median (IQR) ^2^
**Expectations**				
Respect as a Human Being	3.0	5.0	4.7 (0.4)	4.8 (4.5–5)
Respect for Personal Feeling and Time	3.0	5.0	4.4 (0.6)	4.5 (4–5)
Respect for Privacy	1.0	5.0	4.3 (0.9)	4.7 (4–5)
Respect for Autonomy	1.5	5.0	4.4 (0.7)	4.5 (4–5)
**Satisfaction**				
Respect as a Human Being	2.3	5.0	4.5 (0.5)	4.7 (4.2–5)
Respect for Personal Feeling and Time	2.4	5.0	4.4 (0.6)	4.5 (4–4.8)
Respect for Privacy	1.0	5.0	4.2 (0.9)	4.5 (4–5)
Respect for Autonomy	1.0	5.0	4 (0.8)	4 (3.5–5)

^1^ Standard Deviation, ^2^ IQR: Interquartile Range.

**Table 3:** Confirmatory Factor Analysis of the IPDS.

Expectations				Satisfaction			
CFI	TLI	RMSEA	SRMR	CFI	TLI	RMSEA	SRMR
0.92	0.91	0.059	0.069	0.93	0.94	0.049	0.071

CFI: Comparative Fit Index; TLI: Tucker–Lewis Index; RMSEA: Root Mean Square Error of Approximation, SRMR: Standardized Root Mean Squared Residual.

**Table 4:** Correlation coefficient between the dimensions of the IPDS.

**Expectations**				
		Respect for Personal Feeling and Time	Respect for Privacy	Respect for Autonomy
Respect as a Human Being	r	0.63	0.58	0.55
	*p*	<0.001 *	<0.001 *	0.001 *
Respect for Personal Feeling and Time	r		0.50	0.51
	*p*		<0.001 *	<0.001 *
Respect for Privacy	r			0.21
	*p*			0.001 *
**Satisfaction**				
		Respect for Personal Feeling and Time	Respect for Privacy	Respect for Autonomy
Respect as a Human Being	r	0.72	0.64	0.69
	*p*	<0.001 *	<0.001 *	<0.001 *
Respect for Personal Feeling and Time	r		0.55	0.55
	*p*		<0.001 *	<0.001 *
Respect for Privacy	r			0.55
	*p*			<0.001 *

* Statistical significance *p* < 0.001.

**Table 5:** Test–retest reliability of the IPDS (N = 40).

	Expectations			Satisfaction		
Items	ICC	95% CI	*p*	ICC	95% CI	*p*
1	0.8	0.49–0.92	0.001	0.95	0.88–0.98	<0.001
2	0.9	0.75–0.96	<0.001	0.98	0.94–0.99	<0.001
3	0.86	0.65–0.95	<0.001	0.98	0.94–0.99	<0.001
4	0.86	0.65–0.95	<0.001	0.96	0.89–0.98	<0.001
5	0.94	0.86–0.97	<0.001	0.88	0.68–0.95	<0.001
6	0.94	0.84–0.98	<0.001	0.89	0.73–0.96	<0.001
7	0.74	0.34–0.90	0.003	0.84	0.60–0.94	<0.001
8	0.58	−0.06–0.83	0.033	0.76	0.40–0.91	0.001
9	0.97	0.93–0.99	<0.001	0.98	0.96–0.99	<0.001
10	0.93	0.82–0.97	<0.001	0.97	0.93–0.99	<0.001
11	0.85	0.63–0.94	<0.001	0.96	0.89–0.98	<0.001
12	0.7	0.24–0.88	0.006	0.86	0.65–0.95	<0.001
13	0.8	0.49–0.92	0.001	0.78	0.44–0.91	0.001
14	0.77	0.43–0.91	0.001	0.97	0.92–0.99	<0.001
15	0.91	0.78–0.97	<0.001	0.91	0.78–0.97	<0.001
16	0.91	0.77–0.96	<0.001	0.89	0.72–0.96	<0.001
17	0.79	0.80–0.95	0.077	0.78	0.47–0.78	0.110
18	0.79	0.47–0.92	0.001	0.69	0.22–0.88	0.007
19	0.80	0.49–0.92	0.001	0.75	0.23–0.81	0.063
20	0.83	0.58–0.93	<0.001	0.87	0.67–0.95	<0.001
21	0.78	−0.19–0.81	0.055	0.79	0.46–0.92	0.001

CI: Confidence Interval; ICC: Intraclass Correlation Coefficient. Statistical significance was set at *p* < 0.05.

**Table 6:** Internal consistency of the IPDS.

**Expectations**			
Items	Corrected Item-Total Correlation	Cronbach’s Alpha If Item Deleted	Cronbach’s Alpha
Respect as a Human Being			
1	0.64	0.74	0.79
2	0.55	0.77	
3	0.61	0.75	
4	0.50	0.77	
5	0.51	0.77	
6	0.55	0.76	
Respect for Personal Feeling and Time			
7	0.35	0.54	0.71
8	0.50	0.38	
9	0.35	0.52	
14	0.33	0.53	
Respect for Privacy			
19	0.50	0.85	0.79
20	0.74	0.59	
21	0.72	0.63	
Respect for Autonomy			
11	0.42		0.70
12	0.42		
**Satisfaction**			
Respect as a Human Being			
1	0.33	0.80	0.79
2	0.47	0.77	
3	0.49	0.77	
4	0.57	0.77	
5	0.74	0.72	
6	0.77	0.70	
Respect for Personal Feeling and Time			
8	0.38	0.77	0.78
9	0.54	0.75	
10	0.56	0.75	
14	0.47	0.76	
15	0.55	0.75	
16	0.48	0.76	
17	0.56	0.75	
18	0.43	0.77	
Respect for Privacy			
19	0.60	-	0.74
21	0.60	-	
Respect for Autonomy			
11	0.58	-	0.74
12	0.58	-	
Total Cronbach’s alpha for Expectations			0.82
Total Cronbach’s alpha for Satisfaction			0.90

## Data Availability

The data presented in this study are available on request from the corresponding author. The data are not publicly available due to ethical and privacy restrictions.

## Abbreviations

The following abbreviations are used in this manuscript:

HD	Hemodialysis
IPDS	Inpatient Dignity Scale
